# An intervention development for cessation of hookah smoking among Iranian women: study protocol for a systematic and theory-based approach to intervention design

**DOI:** 10.1186/s13722-020-0184-y

**Published:** 2020-02-03

**Authors:** Sakineh Dadipoor, Gerjo Kok, Ali Heyrani, Teamur Aghamolaei, Mohtasham Ghaffari, Amin Ghanbarnezhad

**Affiliations:** 1 Social Determinants in Health Promotion Research Center, Hormozgan Health Institute, Hormozgan University of Medical Sciences, Bandar Abbas, Iran; 2 Department of Work and Social Psychology, School of Psychology and Neuroscience, Maastricht University, Maastricht, The Netherlands; 3 Cardiovascular Research Center, Hormozgan University of Medical Sciences, Bandar Abbas, Iran; 4 Environmental and Occupational Hazards Control Research Center, School of Public Health, Shahid Beheshti University of Medical Sciences, Tehran, Iran

**Keywords:** Hookah, Intervention mapping, Quasi-experimental trial, Smoking, Women

## Abstract

**Background:**

Hookah smoking is an emerging global health issue, especially in Eastern Mediterranean region; and accordingly, women are at the center of this issue as they have a more positive attitude towards hookah smoking. Also, the rate of hookah smoking is increasing at a faster rate among women compared to men. The aim of the present study will be systematically designing and implementing an educational intervention program for hookah smoking cessation among 15 years old women and older in the Iranian southern city of Bandar Abbas.

**Methods:**

We will use intervention mapping methodology for designing the study. Due to the breadth of factors affecting hookah smoking and the complexity of hookah cessation, we will initially conceptualize hookah smoking cessation program as a set of purposeful activities designed to stop the hookah smoking. In the first step (need assessment), the environmental and behavioral factors related to hookah smoking/cessation and the determinants of these factors will be determined by systematic review and local qualitative study. Then, based on the results of the first step, the behavioral and environmental goals of hookah smoking cessation will be identified. In the second step, the practical goals will be determined for each of the behavioral and environmental outcomes; and then, the logic and matrix of change objectives will be designed using the determinants extracted from the previous step. The products of the second step will be the intervention goals. In the third step, theoretical and practical methods affecting each of the intervention goals will be identified. In the fourth step, contents of educational program for hookah cessation will be produced. The fifth step will be about planning to implement the program. In the sixth step, the effectiveness of designed program will be evaluated in a quasi-experimental intervention.

**Discussion:**

Appropriate development and successful implementation of a hookah cessation intervention requires a systematic and theory-based approach. We believe that using Intervention Mapping (IM) as the guiding methodology will make it possible to address complexities of developing an intervention program. Also, reflections on the quasi-experimental research and describing the context and executed methods of implementation would contribute to the development of IM and the knowledge needed for the implementation of program.

*Trial registration* IRCT20190126042494N1, Registered 3.3.2019. https://en.irct.ir/trial/37129.

## Background

Today, the use of hookahs is considered as one of the most important global issues, especially in Arab countries, Turkey, and Iran [1]. Eastern Mediterranean region have the highest prevalence rate of hookah smoking (HS), and this trend has been increasing in the last two decades [2–4].

Social, psychological, cultural, and biological differences between women and men, especially in relation to causes of tendency towards various substances, have made it more obvious that this issue is more acute among women compared to men [5]. Based on women’s perception, social acceptance is higher for hookah than for cigarette [6]; and women have a more positive attitude toward hookah compared to men [7]. Accordingly, the world-wide statistics indicate an increase in the rate of hookah consumption among women compared to men [8, 9].

In Iran, women have more restrictions on the use of cigarettes than hookahs [10]. The results of a widespread survey performed in 2007 showed that 82% of Iranian women prefer smoking hookah than cigarette, and this makes hookah the most popular means of smoking compared to the other ones (pipe, cigarettes, etc.) [11].

In an epidemiological survey, the prevalence of hookah consumption among women in three southern provinces of Iran was estimated to be 16.8% in Sistan, 14.8% in Bushehr, and 10.3% in Hormozgan [12]. This rate is 7% to 8% among women in Eastern Mediterranean region [13], 4% among Lebanese women [14], and 4% among Pakistani women [15].

Considering the complexity of behaviors such as smoking; different models and theories have been developed in health education, which in the situation of appropriately applying, they could result in reducing the rate of harmful behaviors [16]. In smoking cessation interventions, relevant and effective techniques of behavioral change should be used [17]. The designers’ goal will be producing a product from an intervention, which is complex and requires a set of behavioral change techniques. Thus, program designers should be able to present numerous theoretical-practical approaches [17]. On the other hand, one of the main challenges of smoking cessation is the lack of participation of target group in the interventions. Among adults, the mean ratio of participation in intervention programs is approximately 2%. [18]. Therefore, to stop hookah smoking, it seems that several factors such as the use of coherent, ecological, and theoretical-based approach as a roadmap to provide guidelines for designing evidence-based interventions, using relevant and effective techniques of behavioral change, and participation of target population are essential.

Intervention mapping (IM) introduces a series of systematic steps and processes that help health promoters to develop theoretical and evidence-based programs. IM is characterized by three factors as follows: ecological approach, participation of all stakeholders, and the use of theory and evidence. IM describes the process of planning health promotion programs in six steps as follows: (1) assessing the needs; (2) creating the matrix of goals for behavioral change; (3) choosing theory-based approaches and practical strategies; (4) developing the intervention; (5) planning the implementation of program; and (6) planning the evaluation [19].

Most studies conducted on the use of hookah among women have only addressed some aspects such as knowledge and attitude towards smoking [20], prevalence of hookah consumption [21], previous and future educational interventions [16], factors affecting hookah consumption [22], and the relationship between hookah consumption and health consequences [23]. Some of these studies, without identifying important determinants, have tried to select predictors of behavior change from a theory without clearly referring to how behavior may really change. Also, most smoking cessation interventions only focus on individuals, and do not take into account the behavioral and environmental factors. In order to eliminate the above-mentioned limitations, we need a model to put determinants of behavior at the center of intervention design. Regarding, we believe that IM can suit this purpose. Therefore, the main objective of our research will be to systematically design and implement an educational intervention program for hookah cessation among 15 years old women and older in the Iranian southern city of Bandar Abbas in terms of the guide of IM methodology. This paper describes the systematic planning process.

## Methods/design

**Fig. 1:**
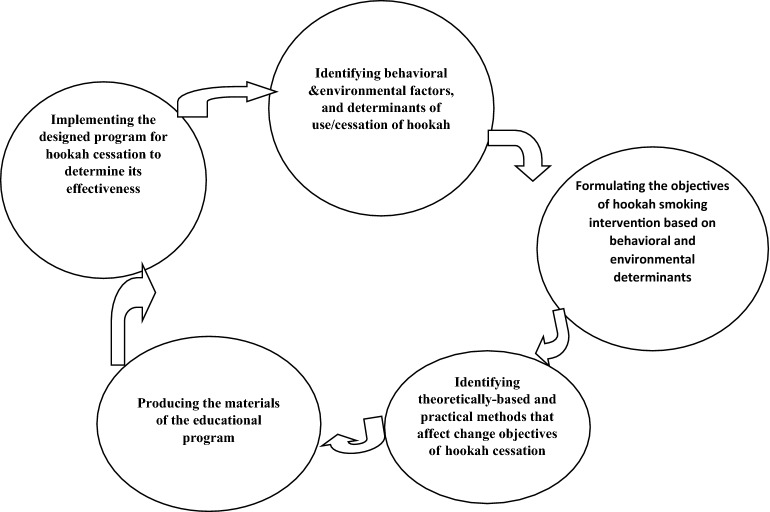
The concept of hookah smoking cessation

To design and direct the study, we will create a research team comprising of 5 experts in health education and promotion, 2 experts in health care management, 2 clinical psychologists, 2 sociologists, and 3 experts in the field of tobacco consumption. Then, the main stages of study will be conceptualized with the aim of designing an educational intervention program for HS cessation (Fig. 1).

### Step 1: Assessing the needs

The purpose of this stage will be identifying the environmental-behavioral factors and determinants affecting the use/cessation of hookah. Often needs assessment requires multiple qualitative/quantitative data sources; therefore, at first a comprehensive review will be conducted on national and international documents and articles related to the factors affecting the use of hookah, then to identify the behavioral-environmental factors and determinants affecting hookah smoking/cessation; a qualitative study with content analysis approach will be conducted. The determinants of hookah smoking/cessation extracted from the qualitative and review studies will be categorized according to the intervention mapping guideline at different levels (individual, interpersonal, organizational, social, and political). The results of systematic review and exploratory study will then be summarized, merged, and analyzed.

#### 1-1: Systematic review of literature

In order to identify behavioral and environmental factors affecting the HS among women and it’s determinants at the individual and interpersonal levels; a systematic review study will be conducted.

##### Search strategy

According to the study objectives, key words related to HS will be selected. Then, search terms will be constructed using the combinations of key words (Table 1). To find relevant literatures, databases such as Web of Science, PubMed, Iranian literature, Elsevier, Embase, Scopus, and Medline as well as Google Scholar and WHO websites will be investigated. A reference search of the grey literature in journals, research abstracts, conference proceedings, and thesis will also be conducted.

**Table 1:** The search terms

	Search term
#1	“Factors”[tiab] OR “affecting”[tiab] “ Risk Factor”[tiab] OR “ Risk Factors”[tiab] OR “Related Factor”[tiab] OR “Related Factors”[tiab] OR “Associated Factor”[tiab] OR “Associated Factors”[tiab]
#2	“waterpipe smoking”[tiab] OR “Waterpipes”,[tiab] Smoking” [tiab] OR “Pipe”[tiab], Smoking Water” [tiab] OR “Pipe Smoking”[tiab] OR, “Water Smoking”[tiab] OR “Waterpipe Smoking” [tiab] OR “Smoking, Waterpipe”[tiab OR “Smoking pipes” [tiab] Hookah Smoking”[tiab], “Smoking, Hookah”[tiab] OR “Hookahs”[tiab] OR “Hookahs” [tiab] OR “Sheesha”[tiab] OR “shisha”[tiab] OR “Shishas”[tiab] OR “Narghiles “[tiab] OR “Narghile”[tiab] OR “Hubble-bubble”[tiab] OR “Hubble” [tiab] OR“narkeela”[tiab] OR “bubble”[tiab] OR “hubby-bubby”[tiab], OR “Gaza[tiab]” OR “argil[tiab]” OR “oriental[tiab] “ OR “boory” [tiab] OR “glase base” [tiab] OR “ghalyan”
#3	“Female”[tiab] OR “woman”[tiab] OR “women”[tiab] OR “Adolescent” [tiab] OR “Adolescence “[tiab] OR “youth” [tiab] OR youth[tiab]

##### Inclusion and exclusion criteria

Studies focusing on the determinants of hookah consumption/cessation with the study population of women will be entered into the study. Articles with the target population of the groups other than women, studies with mix gender sample, papers focused on other types of smoking (such as cigarettes), and studies exploring other factors (e.g., the correlation between HS and health outcomes) will be excluded. Also, articles that have not distinguish between HS and smoking other substances (studies that explored the effect of several substances simultaneously), interventional studies, and papers that explored the correlation between demographic information and health outcomes will be excluded.

##### Screening and data extraction

Initial screening of studies will be performed based on the information existed in their titles and abstracts, and it will be conducted by two independent investigators (SD and TA). If the reviewers have disagreement on a case, the article will be re-evaluated, and if the disagreement persists, a third reviewer (AH) will make the final decision. Full-paper screening will be conducted by the same independent investigators. The data obtained from the articles used in this study will be extracted by two independent authors using standard data extraction tool. The extracted data will contain specific details about relevant data concerning the first author, publication year, purpose of the study, setting, sample size, and determinants of HS among women at intrapersonal, interpersonal, institutional/organizational, social, and political levels.

##### Quality assessment

Papers selected for retrieval will be evaluated by two independent authors for methodological validation before entering the review using 22-item checklist “Strengthening the Reporting of Observational Studies in Epidemiology (STROBE)”, [24]. Articles will be classified as good (scores of 17–22), medium (8–16), and poor (1–7). Then, a critical appraisal of the qualitative studies will be conducted using the checklist for qualitative research with 10 items [25]. In case of arising any disagreements between reviewers, it will be resolved by discussion or a third reviewer. All related qualitative, quantitative, and mixed-method articles as well as scientific publications will be included.

#### 1-2: local exploratory study

*Aim* A local exploratory study with conventional content analysis approach will be conducted to accurately identify the behavioral-environmental factors and determinants involved in the use/cessation of hookah.

##### Study design

The exploratory content analysis will be used in this qualitative study. The determinants of consumption/cessation of hookah in the personal, interpersonal, institutional/organizational, social, and political levels will be extracted by conducting semi-structured interviews with women who had unsuccessful hookah cessation attempt, and also those women who had successful hookah cessation attempt, as well as professional experts in the field of tobacco consumption.

##### Study sample

About 40 women and 20 experts are estimated to participate in this study to identify the determinants of hookah consumption/cessation.

Purposeful sampling using snowball technique will be conducted taking into account the maximum variation in characteristics such as age, occupation, education, and HS status (period of cessation and type of cessation such as successful or unsuccessful). Sampling will be continued until data saturation, when no new data is obtained from the interviews.

##### Inclusion/exclusion criteria

Entry criteria will include the followings: being a native of Bandar Abbas, being a female who smoked hookah at least 7 times a week for 6 consecutive months, having a good experience and understanding of the subject, being an ex-smoker with a history of smoking at least 6 times a week for 6 consecutive months before quitting, and having stopped smoking for more than 6 months. Also, the professional experts will be selected based on the inclusion criteria among those who have extensive information on the topic of study. The ability to communicate and willingness to share information will also be other criteria to enter the study. Exclusion criteria will include the followings: smoking other types of tobacco products, not willing to continue the interview at the time of interview, and the individuals who will admit they cannot help due to inadequate information after hearing the study objectives presented by the interviewer.

##### Data collection

Data collection will take about 10 months (40 interviews with women and 20 interviews with experts). After obtaining ethics approval and a written informed consent from all participants, the interviews will be conducted by SD, after performing two pilot interviews. To ensure the quality of interviews, one or two interview(s) will be conducted per day.

First, participants will answer the demographic questions, and then the interview will be started by asking open core questions. With the advancement of interviews and concurrent analysis of data, the questions will be more detailed. The duration of each interview is estimated to be around 1 h.

##### Interview guide

For the interviews, an initial interview guide will be prepared, which will include two parts as follows: the first part will be related to demographic information and the second part will be related to overt and covert causes of hookah consumption and women’s attitude towards hookah consumption. After the first five interviews, participants’ feedback will be considered to finalize the interview guide; and accordingly, the rest of the interviews will be held. Each interview will be started by asking 5 main questions in the interview guide. In the proceeding, follow up questions will be asked to elaborate on the details. Probe questions will also be used to explore the subject. The primary questions include the follows: How did hookah find its way into your life? How did you end up being a hookah smoker? Why do you smoke hookah? In your opinion, what would turn someone into being a hookah smoker and what would make him/her to continue this habit? Why the rate of hookah consumption is higher among women in Bandar Abbas?

##### Data analysis

All interviews will be audio-taped and transcribed verbatim with participants’ permission, and will be then coded through conventional content analysis method by SD. The interviews will be independently reviewed by SD and TA line-by-line. Then, SD and TA will review all the extracted codes in several meetings, and will discuss and examine the extracted categories and sub-categories. In case of disagreement on the categories and sub-categories, they will try to solve the problem by referring to the initial interviews and reviewing the codes.

In fact, since our purpose of the interview is to discover the behaviors and determinants of hookah consumption, we will be sensitive to these issues at the time of analysis. Therefore, we will carefully read the answers and encode them to find a word or phrase related to the purpose of interview (the interviewee’s speech refers to a behavior or a determinant of hookah smoking). As the work will progress, we will compare the new codes with the previous ones. This may lead to merging, deleting, or changing the codes. Finally, we will categorize the resulting codes into categories, based on their similarities and concepts. Information management will be done using MAXQDA software version 10.

##### Consent, confidentiality and data protection

All participants will receive the invitation and written informed consent forms. Prior to signing of the forms, the researcher will orally explain the purpose of study to them. To ensure the confidentiality of information, no name or surname will be recorded. After transcribing the interviews, the recorded information will be deleted and the interviews’ text will be saved in a computer that only SD will have the access to that. The consent forms will also be kept in a safe place by the SD.

##### Rigor

The researchers will try to strengthen the credibility of findings by taking following ways: (1) Allocating sufficient time to data collection; (2) reporting the findings back to a number of participants to be reviewed by them; (3) sending the data to two colleagues (AH, MG) who have experience in the qualitative research, and based on their comments, the categories and sub-categories will be reviewed and revised; also, to increase the validity of findings, categories, sub-categories, and examples of the codes will be sent to two scholars who are not members of the research team; and (4) Applying maximum variation in selecting interviewees in terms of age, employment, education, hookah consumption status (women who smoke hookah, women who used to smoke hookah), and experienced experts; (5) using interview guide.

We will try to facilitate the transferability of the results by describing the study context, providing a complete description of the participants’ characteristics, the method of data collection, and analysis in systematic steps with an example of participants’ statements, and explaining the barriers and limitations of the study.

#### 1-3: Combining the results to understand the issue of HS

After gathering the results of systematic review and exploratory study, the research team will summarize, integrate, and analyze them. A framework called the Triangulation Protocol (TP) will be used to synthesize diverse information [27]. In this way, at first, the need assessments of the different sections will be entered in a table called the needs assessment table; and then, similar items in each of the need assessment sections will be considered as complementary information and any disagreement will be resolved through discussion and consensus with the research team members. The focus of analysis will be identifying determinants of behavioral-environmental factors [28]. Individual and environmental factors will be prioritized according to their importance and variability during the meeting with the research team members; and behavioral and environmental conditions that are more relevant and changeable will be prioritized for the intervention. Based on the results of need assessments, a logical model of problem (consisting of health, behavioral and environmental factors of HS and its determinants) will be outlined. Once the determinants and behavioral and environmental factors are identified, we can claim that these factors lead people to smoke hookah. In this study, we intend to design and implement an educational intervention program to help people quit HS (and cessation) in a way that targets the determinants and environmental/behavioral factors affecting women’s smoking. Therefore, in the second step, we will formulate clear goals for designing and implementing the required interventions.

### Step 2: Creating the matrix of objectives for behavioral change

In the second step, behavioral and environmental outcomes will be identified for the goals determined in the previous step, and practical goals will be written for the realization of these outcomes. Then, each of the practical goals will be compared with the most important and changeable determinants extracted in the previous step and the matrix of change goals will be created by intersecting practical goals with the determinants.

#### Determining the aims of intervention

At the beginning of this step, we will determine the expected behavioral and environmental outcomes of the educational intervention by utilizing the results obtained in the first step of the study. For example, when we find that spending time with friends who smoke hookah is one of the causes of hookah smoking, then the expected outcomes of our educational intervention should be the following; “individuals can terminate their relationship with hookah consumer friends”. Therefore, any of the behavioral and environmental factors affecting HS in women will be the source of choice for an expected outcome of educational intervention. Then, the expected behavioral and environmental outcomes will be expressed as expected practical goals that are going to be achieved through step-by-step activities for fulfilling behavioral and environmental outcomes. On the other hand, the determinants or root causes of behavioral and environmental factors affecting the use of hookah in women will be cleared in the first step. Therefore, in the second step, by forming a matrix that will include the expected practical goals of the educational intervention and the determinants of behavioral and environmental factors affecting the use of hookah; clear educational goals will be determined to design the intervention program. According to the purpose of this study, two matrixes will be formed for the two levels of individual and interpersonal intervention.

#### Quality control of the matrix of change goals

Validation of practical goals will be as follows: Planners will conduct face-to-face interviews with ten professional experts (trained general practitioners who have completed smoking cessation courses, health education specialists, and experienced psychologists) to confirm the practical objectives. These interviews will be implemented and eventually analyzed with the consent of participants. After receiving the expert opinions, the practical objectives will be reviewed. Also, during the focus group discussion with the potential participants of the intervention program (whose characteristics have been previously described); the practical objectives will be discussed and their views on these objectives will be considered. After presenting the opinions of the experts and the target group, the draft list of practical objectives will be reviewed and finalized in an expert panel meeting.

#### Validation of determinants

The determinants will be determined using the systematic review and local qualitative study described in the first step. An initial list of determinants will be developed and will be then graded based on the relevance (strong association with behavior) and variability (impact of educational intervention on determinants). Also, we will perform a number of ways to prioritize these determinants. First, a broad search will be conducted in all valid databases to relate the determinants with the smoking cessation behavior using related keywords. After prioritizing the determinants based on the literature review and cross-sectional study, the views and opinions of potential participants of the program will be examined through a focused group discussion. The opinions of participants will be applied; and then, the variability, importance, and relevance of determinants of HS will be addressed in an expert panel meeting. We will also conduct a cross-sectional study to more closely explore the determinants of HS, which are described below in detail. After summarizing the comments and results of the cross-sectional study, the determinants will be prioritized and approved.

Validation of the matrix of change goals: The matrix of change goals will be designed based on the opinions and ideas of the experts and potential participants of the program. These goals will be confirmed by the research team during two to three sessions after being edited and reviewed.

The product of second step will be designing the intervention objectives.

### Step 3: Choosing theory-based approaches and practical strategies

Once the change objectives will be identified; we will use IM to identify methods that have sufficient and effective impacts on those objectives. Therefore, we will use appropriate and relevant theoretical and practical methods, which will be determined in a number of ways. First, we will have a comprehensive overview of all theories of health education and health promotion to determine the appropriate theoretical and practical approaches to the determinants. For example, what will be proposed by the theoretical and practical methods to change the knowledge in scientific texts? In the next step, these theoretical and practical approaches will be sent to five health education experts with experience in the field of behavior change theories. After applying the experts’ comments, these methods will be communicated to potential program participants, and in a focus group discussion we will be sure that these methods are in line with the beliefs and culture of the target group. After gathering the opinions of experts and women in the target group, they will be reviewed again at the expert panel meeting; and if necessary, they will be revised.

#### Quality contro**l** of “choosing theory -based approaches and practical strategies”

The theoretical and practical approaches affecting the change objectives will be identified on the basis of empirical evidence, extensive review of behavior change theories, and opinions of experts and potential program participants. Based on the theoretical and practical approaches, the scope, sequence, and context of the program will be determined to some extent, and these will be considered as the products of third step.

### Step 4: Developing the intervention

At this step, the main material of the program will realistically be produced. These will include program content, activity sequences, messages, presentation channels, and scheduling. Before producing the program material, some documents will be designed. These documents, which include the complete project information such as project duration, goals, budget, list of change objectives, and theoretical and practical methods; will be useful guides to produce the program materials. Messages and program materials will be produced based on the designed documents. It should be noted that, budget and time frame will be taken into account in designing the program materials. After performing the initial design of the program, the materials will be reviewed during a meeting with ten potential program participants to ensure that messages and presentation channels are accepted. After applying the audience feedbacks, the research team will discuss the design of the program during an expert panel meeting, and will revise it if needed. Prior to the actual implementation of program, the materials will be examined in a small sample group to ensure that they are sufficiently acceptable and executable.

#### Quality control of the intervention development process

At this stage, the researcher will invite the participants to obtain their views on the components and materials of the program; and based on their views and cultural issues, the materials and components of the program will be revised. At first, five training sessions will be held on theoretical approaches and practical applications for women and those who have influence on them. Each session is estimated to take 90 min and will be held once a week. Materials and components of the program will be reviewed.

### Step 5: Planning the implementation of the program

In this study, the fifth IM step, which involves planning to implement the program, will not be taken in detail until the first evaluation study will be executed. From the beginning of the program, the research team will be composed of a mix of potential program participants and implementers, who will be involved at all stages of the program design. Also, their ideas and comments will be used in all parts of the program. The produced materials will be pre-tested prior to actual large-scale implementation of the program to ensure adequate adoption and implementation of the program.

### Step 6: Planning the evaluation

Evaluating each one of the intended health outcomes, behavioral and environmental determinants, and change objectives of HS will be addressed by designing evaluation questions and taking into account the budget and time frame. In this step, we will review the logical pattern of change and design evaluation questions based on determinants, behavioral and environmental goals, change objectives, and outcomes. We will then evaluate the effectiveness of designed program in a quasi-experimental study. The purpose of quasi-experimental intervention will be evaluating the effectiveness of designed educational intervention on smoking cessation/reduction rate among women.

The target population in this study will be all 15 years old women and older in Bandar Abbas.

#### Sample size

To calculate the sample size in this semi-experimental study, the sampling formula in comparative studies will be used, considering (d = 1, s1 = 2.55 and s2 = 2.38, respectively) based on the findings of similar studies (self-efficacy three months after training in both intervention and control groups in the study of Ahmed Sotoudeh), [26]. Also, considering the errors of α = 0.05 and β = 0.2 (power of 80%), the sample size will be calculated as 96 individuals in each group. However, by taking 10% sample drop into account, the number of samples in each group will be estimated as 106 individuals.

#### Recruitment

The random method will be used for sampling. First, four comprehensive health centers will be randomly selected in Bandar Abbas, from which, two will be randomly assigned to the intervention group and two to the control group. The number of samples from each center will be determined according to the population covered by the center. To select eligible individuals, SD will randomly select a family record belonging to the population covered by the selected comprehensive health centers.

Then, to select other people, the researcher will visit all houses on the right side of the first selected record. After identifying the samples and obtaining informed consent from them, the phone number of all samples will be obtained for correspondence.

#### Inclusion criteria

Smoking hookah for 4 times a week in a duration of at least 6 months before the data collection [27, 28], being fluent in native language, having no history of psychiatric disorder, cardiovascular disease (acute myocardial infarction, unstable angina, severe arrhythmia, and considering the prohibition to use nicotine replacement therapy) [29], not being pregnant or breastfeeding, residing in Bandar Abbas, and giving consent to participate in the study are among the inclusion criteria.

#### Exclusion criteria

Simultaneous use of cigarettes and other tobacco products or drugs, and being absent in more than 20% of the training sessions are among the exclusion criteria.

With regard to the research objectives set out in the previous steps, appropriate training strategies and activities will be selected for the intervention group, and later on, the types of anticipated activities will be introduced to the samples in intervention group in terms of the scheduled program. Then, the training intervention will be implemented based on the previous steps. The number of sessions, duration of each session and intervention, and number of participant in each intervention session will be determined by the research team according to the needs of intervention group. The assessment will be performed based on a pretest that will be taken before the beginning of the intervention, as well as 3، 6, and 12 months after the start of training program.

#### Instrument

The measurement tool will be similar to the final version of questionnaire, which will be designed based on the matrix (change, determinants, and practical objectives). The questionnaire will consist of two parts as follows: demographic information, and change objectives and expected outcomes of the program. The validity and reliability of the questions will be examined by the opinions of a number of experienced experts in this field.

*The measurement of primary outcomes* (1) level of hookah cessation immediately after the intervention, (2) level of hookah cessation within 3 months of the intervention, (3) level of hookah cessation within 6 months of the intervention, and (4) level of hookah cessation within 9 months of the intervention.

*The measurement of secondary outcomes* (1) Comparison of the frequency of hookah consumption before and after the intervention for those who did not stop hookah smoking, (2) point prevalence of study participants (no hookah use during the past 7 days) at the end of the intervention and at 3, 6 and 12 month follow-up, (3) comparison of the number of hookah cessation attempts before and after the intervention, (4) participants’ tendency to stop hookah 6 months after the intervention, (5) rate of cessation as a result of intervention among the study participants after 6 months, and (6) rate of cessation as a result of intervention among the study participants after 12 months.

#### Duration of the intervention

The implementation phase of intervention will be completed in a duration of 12 months. The women will be recruited and trained for a period of 3 months, and will receive followed up for another 12 months.

#### Statistical analysis

After data collection, the data will be analyzed by SPSS-19 software using descriptive statistics (frequency and percentage, mean and standard deviation), independent and paired t-test, path analysis, and logistic regression analysis. The p < 0.05 will be considered as statistically significant.

#### Ethical consideration

Ethical approval was received for this study from the National Institute for Medical Research Development Grant No. 983514. The trial was registered in the Iranian Trial Registry (IRCT20190126042494N1).

To introduce the researcher to the study environment; a formal letter will be obtained from the university’s research deputy. In the qualitative section, before conducting the interviews, appropriate relationship will be established by the interviewee (introducing the researcher, educational degree, aims of the study, and the reason for selecting the interviewee). Then, A written consent will be obtained from the participants for audio recording. They will be also assured about the confidentiality of their information. In the intervention section and prior to the sampling, the researcher will submit the formal letter, and then introduce herself and explain the aims of study to the women in target group in a clear manner. Then, a written consent will be obtained from the participants and emphasis will be made on the voluntarily participation in the study. Participants will also be assured that their information will remain confidential with the researcher.

## Discussion

In this study, we will use IM as a systematic and theory-based approach to design and develop an effective intervention for hookah cessation among 15 years old women and older in Iranian southern city of Bandar Abbas.

Other studies have also shown that IM can be a useful tool to ensure the effectiveness of interventions developed for cigarette smoking cessation [17, 18]. In this study, we believe that we can develop an effective intervention, because factors affecting the use and quitting of hookah both at the individual and environmental levels can be identified by qualitative interviews with women and experienced professionals in this context. Possibly, interventions that consider individual factors as well as environmental factors can be more effective. According to the researchers’ knowledge, so far, no study has been conducted on the factors affecting the cessation of hookah among women living in southern city of Bandar Abbas, Iran. Therefore, it is necessary to investigate these factors to design an effective intervention in this regard. In the first step of the study, these factors will be reviewed during a need assessment. Then, various theoretical and relevant methods will be used to affect the determinants of hookah cessation. In this regard, research has shown that tobacco cessation interventions should use appropriate and effective behavioral techniques [30]. Also, interventions that use the target group’s involvement in the design and implementation of the program will be more successful compared to non-participatory interventions. In this area of research, one of the main challenges of tobacco cessation is the lack of participation of target group in the intervention [18]. The main outcome of this protocol will be designing an intervention, in which the behavioral and environmental factors of HS and effective determinants of hookah cessation, as well as related theoretical and practical methods of intervention design will be identified and fully considered.

## Strengths and limitations of the study

The design of the present intervention in terms of the IM will have a number of strengths, including the use of a bottom-up approach at all stages, and using the participants’ perspective as well as stakeholders in the design of program. At the need assessment stage, we will make sure that potential stakeholders will be involved in the design of program to ensure adoption and implementation of the program. One of the limitations of this study may be the lack of predicting the budget (cost effectiveness) of this intervention; however, its implementation cost will be measured at the end of the intervention. Biochemical validation will not be used to confirm WTS status. However, participants will be assured of the confidentiality of their answers and personal information.

## Data Availability

Data sharing is not applicable to this article as no datasets are generated or analyzed during the current study.

## Abbreviations

HS: Hookah Smoking
IM: Intervention Mapping

## References

[1] Mosharraf S, Allahdadin M, Reyhani M (2019). Comparison of adverse pregnancy outcomes between hookah and non-smoking women. J Midwifery Reprod Health..

[2] Danaei M, Jabbarinejad-Kermani A, Mohebbi E, Momeni M (2017). Waterpipe tobacco smoking prevalence and associated factors in the southeast of Iran. Addict Health..

[3] Jawad M, Charide R, Waziry R, Darzi A, Ballout RA, Akl EA (2018). The prevalence and trends of waterpipe tobacco smoking: a systematic review. PLoS ONE.

[4] Tucktuck M, Ghandour R, Abu-Rmeileh NM (2018). Waterpipe and cigarette tobacco smoking among Palestinian university students: a cross-sectional study. BMC Public Health..

[5] Sohrab Zade M, Parnian L (2015). Qualitative studies on smoking hookah among girls and young women (case study: Shiraz city). Women Dev Polit..

[6] Baheiraei A, Sighaldeh SS, Ebadi A, Kelishadi R, Majdzadeh R (2015). Factors that contribute in the first hookah smoking trial by women: a qualitative study from Iran. Iran J Public Health..

[7] Eshah NF, Froelicher ES (2018). Knowledge, attitudes, beliefs and patterns of waterpipe use among Jordanian adults who exclusively smoke waterpipes. Eur J Cardiovasc Nurs..

[8] Shearston JA, Park SH, Lee L, Oshinsky C, Sherman S, Weitzman M (2016). Increasing hookah use among adolescent females in the US: analyses from the 2011–2014 National Youth Tobacco Survey (NYTS). Tob Prev Cessat..

[9] Daou KN, Bou-Orm IR, Adib SM (2018). Factors associated with waterpipe tobacco smoking among Lebanese women. Women Health.

[10] Baheiraei A, Sighaldeh SS, Ebadi A, Kelishadi R, Majdzadeh SR (2015). Psycho-social needs impact on hookah smoking initiation among women: a qualitative study from Iran. Int J Prev Med..

[11] Meysamie A, Ghaletaki R, Haghazali M, Asgari F, Rashidi A, Khalilzadeh O (2009). Pattern of tobacco use among Iranian adult population: results of the national survey of risk factors of non-communicable diseases (SuRFNCD-2007). Tob Control..

[12] Nemati S, Rafei A, Freedman ND, Fotouhi A, Asgary F, Zendehdel K (2017). Cigarette and water-pipe use in Iran: geographical distribution and time trends among the adult population; a pooled analysis of national STEPS surveys, 2006–2009. Arch Iran Med..

[13] Azab M, Khabour OF, Alzoubi KH, Anabtawi MM, Quttina M, Khader Y (2012). Exposure of pregnant women to waterpipe and cigarette smoke. Nicot Tob Res..

[14] Chaaya M, Jabbour S, El-Roueiheb Z, Chemaitelly H (2004). Knowledge, attitudes, and practices of argileh (water pipe or hubble-bubble) and cigarette smoking among pregnant women in Lebanon. Addict Behav.

[15] Khan MT, Hashmi S, Zaheer S, Aslam SK, Khan NA, Aziz H (2015). Burden of waterpipe smoking and chewing tobacco use among women of reproductive age group using data from the 2012–13 Pakistan demographic and health survey. BMC Public Health..

[16] Setoudeh A, Tahmasebi R, Noroozi A (2016). Effect of education by health volunteers on reducing water-pipe use among women in Bushehr: an application of health belief model. J Hayat..

[17] Brendryen H, Kraft P, Schaalma H (2010). Looking inside the black box: using intervention mapping to describe the development of the automated smoking cessation intervention ‘happy ending’. J Smok Cessat..

[18] Dalum P, Schaalma H, Kok G (2011). The development of an adolescent smoking cessation intervention—an intervention mapping approach to planning. Health Educ Res.

[19] Kok G, Peters LW, Ruiter RA (2017). Planning theory-and evidence-based behavior change interventions: a conceptual review of the intervention mapping protocol. Psicologia: Reflexão e Crítica.

[20] Iqbal N, Irfan M, Ashraf N, Awan S, Khan JA (2015). Prevalence of tobacco use among women: a cross sectional survey from a squatter settlement of Karachi, Pakistan. BMC Res Notes.

[21] Baheiraei A, Mirghafourvand M, Nedjat S, Mohammadi E, Charandabi SMA (2012). Prevalence of water pipe use and its correlates in Iranian women of reproductive age in Tehran: a population-based study. Med Princ Pract..

[22] Baheiraei A, Sighaldeh SS, Ebadi A, Kelishadi R, Majdzadeh R (2015). The role of family on hookah smoking initiation in women: a qualitative study. Glob J Health Sci..

[23] Nematollahi S, Mansournia MA, Foroushani AR, Mahmoodi M, Alavi A, Shekari M (2018). The effects of water-pipe smoking on birth weight: a population-based prospective cohort study in southern Iran. Epidemiol Health..

[24] Von Elm E, Altman DG, Egger M, Pocock SJ, Gøtzsche PC, Vandenbroucke JP (2007). The strengthening the reporting of observational studies in epidemiology (STROBE) statement: guidelines for reporting observational studies. PLoS Med..

[25] Lockwood C, Munn Z, Porritt K (2015). Qualitative research synthesis: methodological guidance for systematic reviewers utilizing meta-aggregation. Int J Evid Based Healthc..

[26] Sotodeh A, Tahmasebi R, Noroozi A (2016). Application of health belief model to predict factors of nicotine dependece among water pipe smoking women in 2015. J Health..

[27] Mojahed K, Navidian A (2018). The effect of motivational interviewing on self-efficacy to quit hookah smoking in pregnant women. J Hayat..

[28] Jabbour S, El-Roueiheb Z, Sibai A (2003). Nargileh (water-pipe) smoking and incident coronary heart disease: a case-control study. Ann Epidemiol.

[29] Hekmatpoue D, Ouroji M, Shamsi M (2013). Effect of educational program bases on transtheoretical model constructs on cognitive and behavioral processes for smoking cessation. J Urmia Nurs Midwifery Fac.

[30] Michie S, Atkins L, West R (2014). The behavior change wheel: a guide to designing interventions.

